# Microstructure Evolution and Mechanical Properties of Wire Arc Additively Manufactured DSS2209 Duplex Stainless Steel

**DOI:** 10.3390/ma18174066

**Published:** 2025-08-30

**Authors:** Jian Sun, Liang Liu, Long Zhang, Jun Hong, Feihong Liu, Dongsheng Wang, Fei Zhou, Youwen Yang

**Affiliations:** 1 Key Laboratory of Construction Hydraulic Robots of Anhui Higher Education Institutes, Tongling University, Tongling 244061, China; 13683x@tlu.edu.cn (J.S.); yangyouwen@jxust.edu.cn (Y.Y.); 2 School of Metallurgical Engineering, Anhui University of Technology, Ma’anshan 243002, China; 3 Technical Department, Anhui Highly Precision Casting Co., Ltd., Ma’anshan 238100, China; hongjun@highly-ahhl.cc (J.H.); zhoufei@highly-ahhl.cc (F.Z.)

**Keywords:** wire arc additive manufacturing, 2209 duplex stainless steel, microstructure evolution, mechanical properties, thermal gradient, interlayer thermal cycling

## Abstract

This study investigates the microstructure evolution and mechanical properties of DSS2209 duplex stainless steel fabricated via cold metal transfer wire arc additive manufacturing (CMT-WAAM). The as-deposited thin-wall components exhibit significant microstructural heterogeneity along the build height due to thermal history variations. Optical microscopy, SEM-EDS, and EBSD analyses reveal distinct phase distributions: the bottom region features elongated blocky austenite with Widmanstätten austenite (WA) due to rapid substrate-induced cooling; the middle region shows equiaxed blocky austenite with reduced grain boundary austenite (GBA) and WA, attributed to interlayer thermal cycling promoting recrystallization and grain refinement (average austenite grain size: 4.16 μm); and the top region displays coarse blocky austenite from slower cooling. Secondary austenite (γ_2_) forms in interlayer remelted zones with Cr depletion, impacting pitting resistance. Mechanical testing demonstrates anisotropy; horizontal specimens exhibit higher strength (UTS: 610 MPa, YS: 408 MPa) due to layer-uniform microstructures, while vertical specimens show greater ductility (elongation) facilitated by columnar grains aligned with the build direction. Hardness ranges uniformly between 225–239 HV. The study correlates process-induced thermal gradients (e.g., cooling rates, interlayer cycling) with microstructural features (recrystallization fraction, grain size, phase morphology) and performance, providing insights for optimizing WAAM of large-scale duplex stainless steel components like marine propellers.

## 1. Introduction

Duplex stainless steel (DSS) is a type of steel featuring a microstructure with closely balanced or equal proportions of austenitic and ferritic phases, extensively employed in industries including shipbuilding, petrochemical engineering, and medical device manufacturing [1,2,3]. In recent years, owing to its exceptional specific strength and corrosion resistance, duplex stainless steel has exhibited a growing trend to replace conventional bronze-based alloys in the manufacturing of medium-to-large marine propellers [4]. Generally speaking, marine propellers are usually manufactured by casting, which inevitably involves processes such as mold manufacturing, material melting, melt casting, and computerized numerical control (CNC) machining, which has a long production cycle, high cost, and is prone to casting defects and product deformation and other quality problems [5,6,7]. Consequently, the utilization of additive manufacturing (AM) processes for propeller mold fabrication has garnered significant industrial attention.

Based on the material supply methodology, additive manufacturing technologies are categorized into three primary modalities: powder bed fusion [8], powder-fed deposition, and wire-fed deposition. Crucially, powder bed fusion processes inherently require dedicated workspace for powder containment, consequently imposing build volume constraints on powder-bed techniques such as Selective Laser Melting (SLM) [9,10] and Selective Laser Sintering (SLS) [11]. The relatively low production efficiency of powder bed fusion processes has driven the emergence of wire arc additive manufacturing (WAAM)—a wire-fed deposition technique—as a high-throughput alternative for large-scale industrial applications. Wire arc additive manufacturing employs an electric arc as an energy beam to melt metallic wire, fabricating components through layer-by-layer deposition. This process demonstrates exceptional productivity, streamlined workflows, cost efficiency, and near-net-shape material utilization, while producing components with densely uniform microstructures and superior integrated performance [12].

Current research on wire arc additive manufacturing predominantly focuses on modulating fundamental process parameters—such as welding voltage, current, and novel arc deposition strategies—to quantify their effects on the morphological characteristics and mechanical performance of fabricated components. Wittig et al. [13] studied the effect of arc energy on the microstructure evolution of alloys fabricated by cold metal transfer-wire arc additive manufacturing (CMT-WAAM) and found that the ferrite content showed a decreasing trend with the increase in arc energy. Eriksson et al. [14] investigated the microstructure evolution of DSS 2760 fabricated by CMT-WAAM at different heat inputs and found that the ferrite content varied from 15% to 27% with different heat inputs and positions, and the tensile strength decreased with increasing heat input. Wu et al. [4] found that the variable polarity cold metal transfer (VP-CMT) mode was effective in reducing the tensile strength of DSS 2209 in the deposition and redeposition zones. With the same wire feed and travel speed, VP-CMT can produce higher strength DSS parts than normal CMT. Pranav et al. [15] successfully fabricated multilayer ER2594 with reasonable ferrite and austenite contents using the CMT-WAAM method and obtained excellent mechanical properties: yield strength (YS) of 632 ± 13 MPa, ultimate tensile strength (UTS) of 855 ± 11 MPa, and elongation of 44.3 ± 11%.

While substantial research exists on the microstructure and mechanical properties of wire arc additively manufactured (WAAM) 2209 duplex stainless steel (DSS), investigations into cross-section-dependent microstructural variations across different build positions remain limited. This knowledge gap is critical because such spatial heterogeneities fundamentally govern material anisotropy. Given that understanding microstructural evolution during additive manufacturing is essential for optimizing material composition and processing parameters, this study employs 2209 DSS feedstock and cold metal transfer (CMT) technology to systematically characterize microstructural evolution and performance correlations.

## 2. Experimental

**Figure 1:**
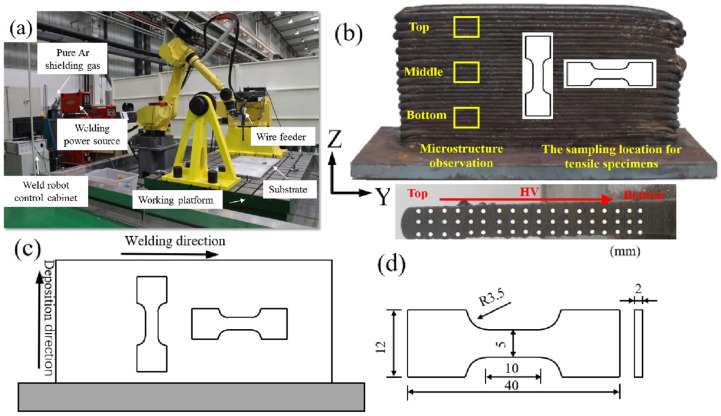
Equipment and physical drawings: (**a**) CMT site diagram; (**b**) physical drawing of 2209 DSS thin-wall assembly by WAAM; (**c**) schematic of thin-wall assembly tensile sample position; (**d**) tensile sample dimensions.

As illustrated in Figure 1a, the CMT-WAAM system (the CMT-WAAM system is an additive manufacturing platform built on FANUC robotic arm (FANUC Corporation, Shinno Village, Yamanashi Prefecture, Japan) and Fronius power supply (Fronius International GmbH, Pettenbach, Austria)) employed in this study comprises a robotic arm, a MIG welding power source, and an automatic wire feeder. The deposition utilized 1.2 mm diameter ER2209 welding wire onto a Q345 hot-rolled steel substrate (250 mm × 110 mm × 8 mm) (Baowu steel group corporation limited, Shanghai, China). Prior to deposition, the substrate was wire-brushed to metallic brightness and degreased with acetone. The cleaned substrate was then mounted on a preheating platform using thermally conductive adhesive. Shielding gas consisted of pure argon (99.99%) at 20 L/min flow rate, with 30 s interlayer cooling intervals. Key processing parameters included 5 m/min travel speed, 5.5 m/min wire feed speed, 4 Hz sinusoidal oscillation frequency at 4 mm amplitude, 0.5 s dwell time, average welding voltage of 19 V, welding current of 90 A, and constant wire orientation, and the gun’s inclination is neutral and heat input *Q* is approximately 16.4 J/mm. Figure 1b shows the as-fabricated 2209 DSS component with elemental composition detailed in Table 1. The extraction location and dimensions of tensile specimens are presented in Figure 1c,d, with specimen axes defined such that length/thickness/height directions correspond to Y/X/Z, respectively.

Samples were sequentially ground using abrasive papers until achieving a scratch-free, mirror-like surface under optical microscopy inspection. Following polishing, surfaces were cleaned by ethanol spraying and dried with compressed air. Etching was performed using an aqueous solution of ferric chloride in hydrochloric acid (5 g FeCl_3_ + 50 mL HCl + 50 mL H_2_O) for 15 s. After etching, specimens were rinsed with deionized water and ethanol, then dried thoroughly. Microstructural examination was conducted using an OLYMPUS BX51 optical microscope (OM) (OLYMPUS Corporation, Tokyo, Japan). The microstructure and elemental composition of WAAM 2209 DSS specimens were characterized using a NANO SEM430 field-emission scanning electron microscope (SEM) (FEI Company, Hillsboro, OR, USA) equipped with energy-dispersive X-ray spectroscopy (EDS). For electron backscatter diffraction (EBSD) analysis, specimens were ground and polished to a scratch-free surface, followed by ultrasonic cleaning in acetone to remove surface contaminants. Electrolytic polishing was then performed using a 10% oxalic acid solution at 15 volts for 50 s. The prepared specimens were examined in a SIGMA 500 scanning electron microscope (Carl Zeiss AG, Oberkochen, Germany) with a 500 μm × 500 μm scan area and 1.5 μm step size, with acquired data processed using Channel 5 software. Tensile testing was conducted on a WNW-30G universal testing machine (Jinan Times Gold Testing Machine Co., Ltd., Jinan, China) at a constant crosshead speed of 1 mm/min. Fracture surfaces of tested specimens were subsequently examined using a JSM-6490LV scanning electron microscope (JEOL Ltd., Tokyo, Japan).

## 3. Results and Discussion

### 3.1. Microstructure Characterization

#### 3.1.1. Metallographic Microstructure

**Figure 2:**
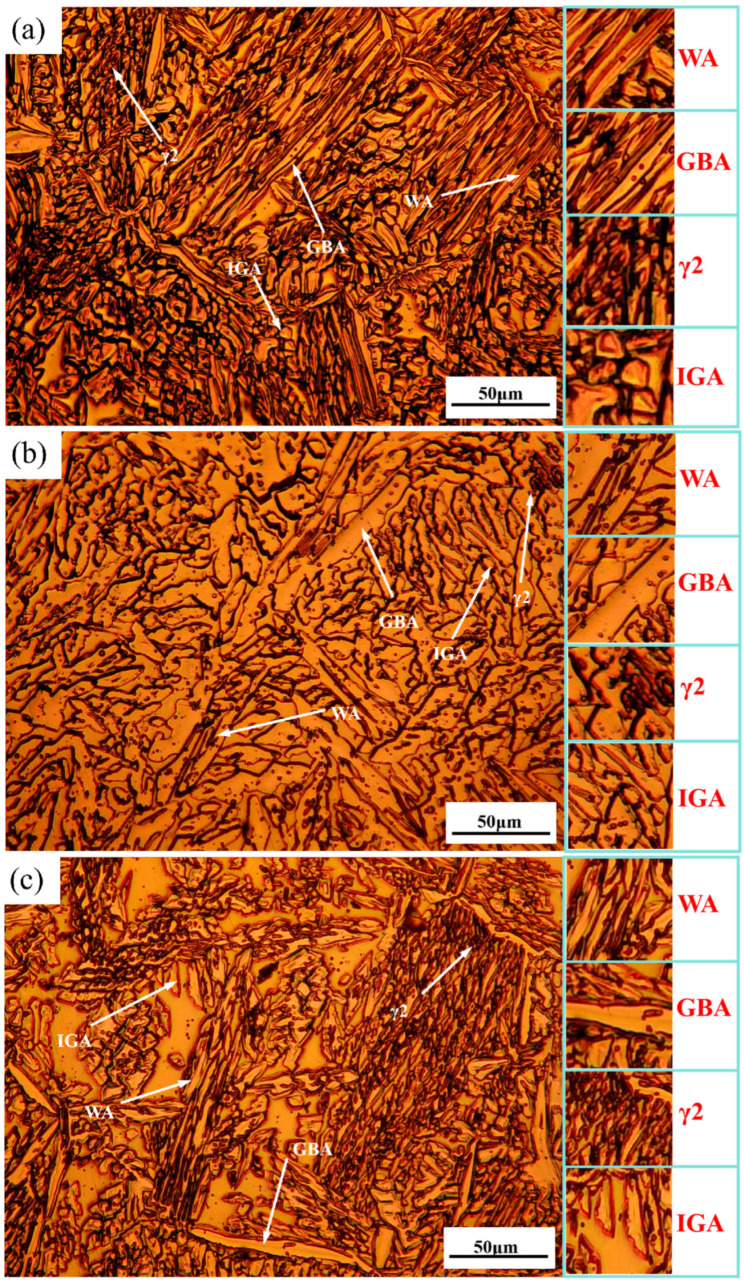
Microstructure of WAAM 2209 DSS: (**a**) the bottom region; (**b**) the middle region; (**c**) the top region.

Figure 2 presents optical microscopy (OM) images of distinct regions in WAAM 2209 DSS. The bottom microstructure predominantly features elongated blocky austenite with Widmanstätten austenite (WA) exhibiting near-parallel alignment, as shown in Figure 2a. This morphology arises from the substrate’s heat sink effect during deposition, which accelerates cooling rates and establishes steep thermal gradients—conditions promoting directional grain growth. Conversely, the middle region is characterized by equiaxed blocky austenite with diminishing grain boundary austenite (GBA) and WA content (Figure 2b). Here, restricted heat dissipation to ambient air results in slower cooling rates and reduced thermal gradients. This thermal environment permits extended austenite precipitation and growth from ferrite, enabling adjacent austenite grains to coalesce into blocky structures. The top microstructure (Figure 2c) similarly displays dominantly blocky austenite but remains unaffected by subsequent thermal inputs, resembling the bottom region in phase distribution yet differing morphologically due to distinct thermal histories.

Four distinct austenite morphologies are observed in the bottom, middle, and top regions: grain boundary austenite (GBA), Widmanstätten austenite (WA), intragranular austenite (IGA), and secondary austenite (γ_2_). Among these, GBA, WA, and IGA constitute primary austenite formed during solid-state transformation following molten pool solidification. Their evolution initiates with ferrite precipitation from the high-temperature liquid phase, which subsequently transforms into primary austenite upon cooling [16,17,18]. At prior ferrite grain boundaries—characterized by disordered atomic arrangements and elevated free energy—austenite preferentially nucleates. GBA requires minimal undercooling, initiating nucleation and growth at elevated temperatures [19]. As substantiated by Kaar et al. [20], high boundary energy drives preferential austenite precipitation at these sites. With progressive cooling, GBA proliferation reduces available nucleation sites, prompting the formation of WA: slender, plate-like austenite grains extending inward from grain boundaries into the ferritic phase. Rapid cooling suppresses WA development by inhibiting sufficient nucleation/growth time. Comparatively, IGA demands greater undercooling than GBA/WA, forming at lower temperatures [21]. IGA nucleation occurs preferentially within ferrite domains exhibiting higher Ni/N but lower Cr/Mo concentrations, with its limited grain growth—constrained by these elemental gradients—resulting in finer microstructures [22,23]. Conversely, γ_2_ forms during subsequent-layer reheating in wire arc additive manufacturing, where interlayer thermal cycling facilitates ferrite-to-γ_2_ transformation. This phase develops as intragranular or intergranular γ_2_, exhibiting significantly smaller grain sizes than primary austenite. Notably, intragranular austenite formation at ferrite matrix interfaces is likewise attributable to reheating effects from successive deposition passes.

#### 3.1.2. SEM Microstructure and Morphology with EDS Energy Spectrum Analysis

**Figure 3:**
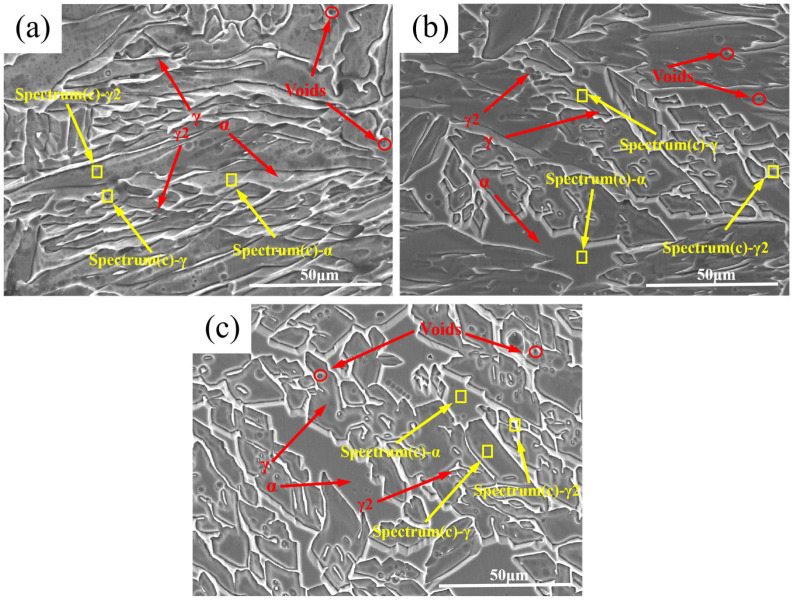
SEM microstructure morphology of WAAM 2209 DSS. (**a**) the top region; (**b**) the middle region; (**c**) the bottom region.

Figure 3 displays the scanning electron microscopy (SEM) microstructures and morphologies of different regions in wire-arc additively manufactured 2209 duplex stainless steel. The lath-like or blocky microstructural features correspond to austenite phases, while the matrix microstructure consists of ferrite. Secondary austenite is observed in certain sections of the microstructure, whereas other areas contain minor porosity. This defect formation originates from the numerous interlayer bonding regions characteristic of WAAM-deposited components, where oxidation films develop on the deposited layer surfaces during molten metal solidification upon exposure to atmosphere. This phenomenon predisposes interlayer zones to defect formation, particularly porosity.

Elemental analysis data from EDS point scans across different regions are presented in Table 2. The elemental compositions in the top, middle, and bottom regions exhibit similar distributions within austenite, ferrite, and γ_2_ phases, with no detectable chromium nitrides or σ-phase present in the microstructure. However, compositional differences exist between γ_2_ and austenite at identical locations: austenite shows higher Cr but lower Ni content compared to γ_2_. This phenomenon primarily arises from differential partitioning behavior and diffusion kinetics of Cr and Ni across phases. At sufficiently slow cooling rates, γ_2_ precipitates from ferrite and accumulates in the interlayer remelted zones of WAAM deposits. Subjected to interlayer thermal cycling, these zones undergo recrystallization which accelerates Cr/Ni partitioning within ferrite. This enables near-complete elemental segregation, where Ni diffuses toward dislocation nucleation sites while Cr and Ni undergo counter-directional partitioning. Consequently, γ_2_ develops reduced Cr and enriched Ni concentrations. Complementary analysis further reveals that ferrite contains higher Cr/Mo but slightly lower Ni levels relative to austenite in both phases. This phenomenon is attributed to the distinct effects of different alloying elements on ferrite and austenite; Cr and Mo, acting as ferrite-stabilizing elements that contract the austenite phase field, consequently partition preferentially into ferrite. Conversely, Ni, functioning as an austenite-stabilizing element that expands the austenite phase region, tends to concentrate predominantly in austenite.

Furthermore, in duplex stainless steels, compositional variations in secondary austenite γ_2_ critically influence microsegregation behavior. γ_2_ forms during δ-ferrite decomposition and inherits Cr/Mo-rich composition from δ-phase due to sluggish diffusion kinetics. This results in Cr depletion at γ_2_/δ interfaces, reducing pitting resistance, while increasing intragranular Mo segregation, which promotes σ-phase nucleation. Concurrently, it lowers N solubility, further increasing the risk of Cr_2_N precipitation. A γ_2_ content exceeding 10% exacerbates elemental partitioning, reducing the critical pitting temperature (CPT) by 8–12 °C and increasing crack sensitivity. Rapid cooling and nitrogen alloying effectively suppress γ_2_ formation and microsegregation [23,24].

#### 3.1.3. EBSD Analysis

**Figure 4:**
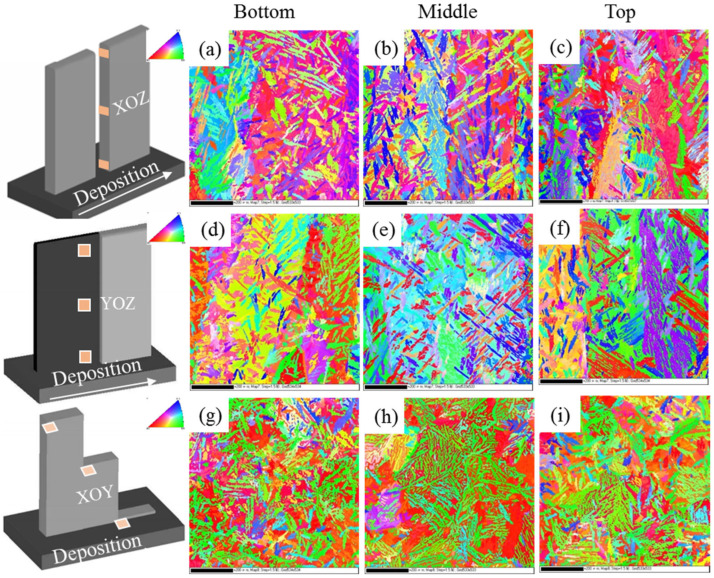
IPF-Z of (**a**–**c**) XOZ-EBSD, (**d**–**f**) YOZ-EBSD, (**g**–**i**) XOY-EBSD.

Figure 4 shows the inverse pole figure of different regions of the WAAM 2209 DSS. From Figure 4, it can be seen that there are differences in crystal orientation at different heights, which suggests that the deposition process is inhomogeneous and there are significant differences in microstructure. However, as shown in Figure 4g–i, an inverse pole figure (IPF-Z) pattern was observed along the Z-direction on the XOY plane of the specimen. In the EBSD maps, the majority of austenite grains exhibit green coloration, indicating a predominant <101>//Z texture orientation, while most ferrite grains appear red, signifying a dominant <001>//Z texture. This selective crystallographic orientation along the build direction suggests texture evolution during grain growth, which is associated with the downward heat dissipation pattern and the columnar growth behavior of austenite grains.

**Figure 5:**
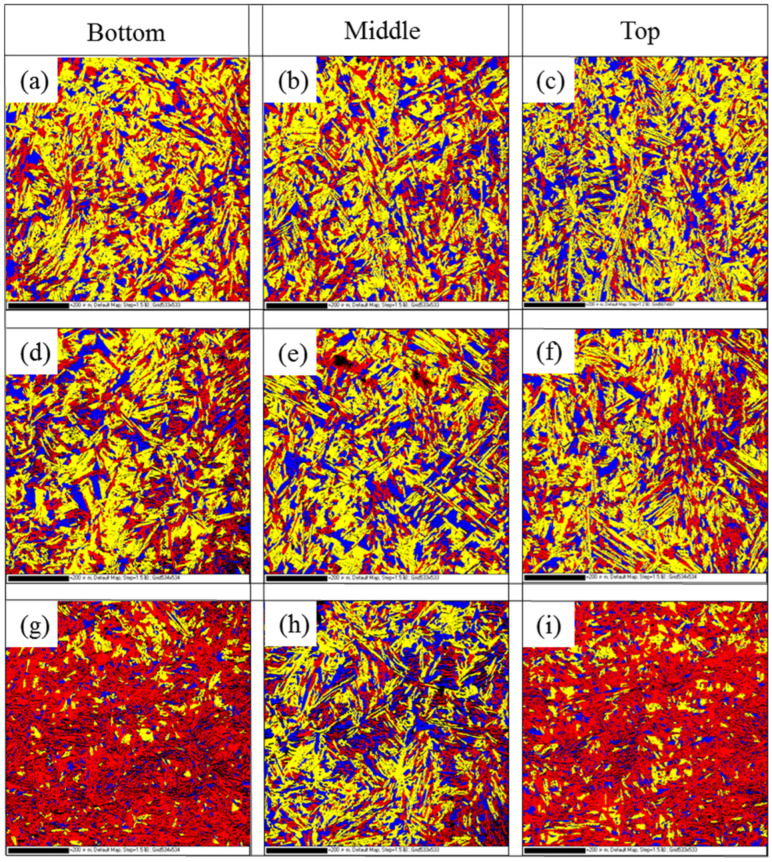
Recrystallization diagrams at different region: (**a**–**c**) XOZ section; (**d**–**f**) YOZ section; (**g**–**i**) XOY section.

Figure 5 shows the recrystallization diagrams of different regions of WAAM 2209 DSS, with blue grains representing recrystallized grains, yellow grains representing substructured grains, and red grains representing deformed grains. It can be seen that the recrystallization maps of the XOZ section and YOZ section show no significant differences. However, the differences are more pronounced in the XOY section, where the lower and middle regions contain more deformed grains than the middle region. The recrystallization fractions at different positions were measured using Channel 5 software, and the results are presented in Table 3.

Based on the statistical results presented in Table 3, it can be observed that, as a whole, the middle region exhibits a higher proportion of recrystallized grains compared to the top and bottom regions. This phenomenon is attributed to the interlayer thermal cycling effect, which prolongs the duration of microstructural exposure to recrystallization temperatures in the middle region, thereby increasing the number of recrystallized grains. Consequently, the final as-deposited section (top region) shows an elevated proportion of recrystallized grains. The microstructure at the bottom resembles that of the substrate. Although also subjected to interlayer thermal cycling, partial heat dissipation into the substrate reduces thermal exposure duration during recrystallization. This results in a lower proportion of recrystallized grains in the bottom microstructure compared to the middle region. During the wire arc additive manufacturing process, the microstructures at the bottom and middle regions have substantially cooled and solidified. Although the top region is still influenced by heat dissipation from the middle microstructure and subjected to interlayer thermal cycling—which increases recrystallized grain count—its cooling rate remains comparable to typical process conditions. Consequently, the proportion of recrystallized grains in the top microstructure is lower than that observed in the bottom and middle regions.

**Figure 6:**
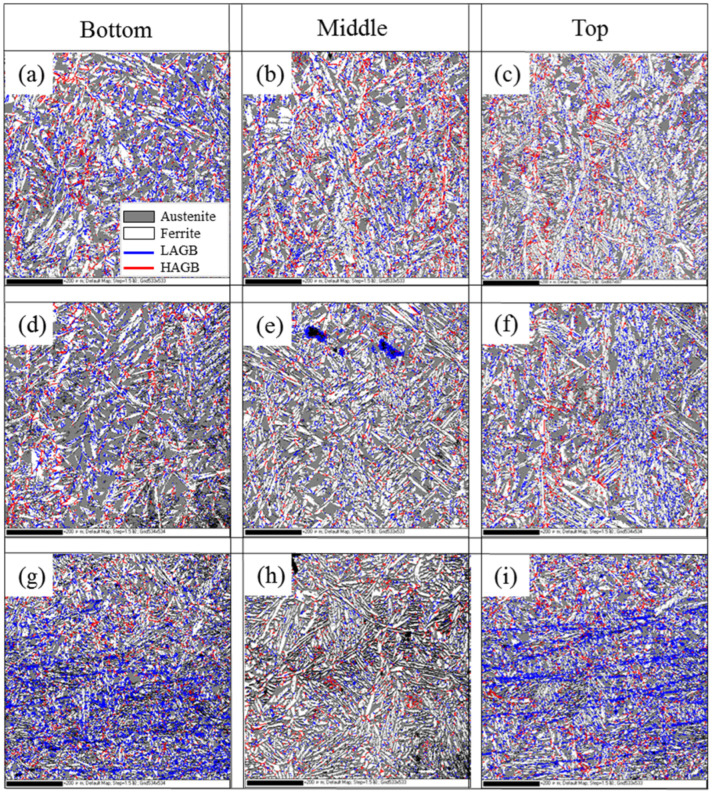
Size-angle grain boundary maps at different region: (**a**–**c**) XOZ section; (**d**–**f**) YOZ section; (**g**–**i**) XOY section.

Figure 6 displays the grain boundary misorientation maps for different regions of the duplex stainless steel. Statistical results from Table 3 indicate that low-angle grain boundaries (LAGBs) predominantly characterize the microstructure. Within austenite grains, the misorientation distribution shows minimal variation between XOZ and YOZ sections. However, in the XOY section, the middle region exhibits a reduced fraction of LAGBs compared to the bottom and top regions. This phenomenon may correlate with interlayer thermal cycling effects during rapid WAAM deposition. According to Reference [25], the interlayer thermal effects in wire arc additive manufacturing prolong recrystallization duration and promote grain growth. During this process, LAGBs progressively absorb dislocations within the microstructure, transforming into high-angle grain boundaries (HAGBs). This mechanism reduces the LAGB fraction, particularly pronounced in the middle region. The distribution trends and underlying causes of HAGBs and LAGBs in ferrite resemble those in austenite. However, since ferrite primarily precipitates from the high-temperature liquid phase and subsequently transforms to austenite during cooling (where austenite nucleates and grows), ferrite requires higher energy to form HAGBs. Consequently, it exhibits a lower HAGB percentage compared to austenite.

The average grain sizes of the different regions of WAAM 2209 DSS were calculated using the average grain size calculation module that comes with Channel 5, as shown in Table 3. The average grain sizes of bottom, middle, and top austenite were 4.5 μm, 4.2 μm, and 5.0 μm, respectively, while those of bottom, middle, and top ferrite were 3.4 μm, 3.2 μm, and 3.9 μm, respectively. Since average grain size is inversely proportional to cooling rate [26,27], variations in cooling rates across different regions induce corresponding changes in grain size. During wire arc additive manufacturing, the top microstructure—being the last-deposited region—experiences cooling rates comparable to conventional conditions despite heat dissipation effects from the middle region, resulting in its slowest cooling rate and thus the coarsest grain size. Conversely, while the middle microstructure undergoes moderately slow cooling due to bidirectional heat dissipation from underlying and overlying deposits, prolonged exposure to interlayer thermal cycling extends its recrystallization duration. This significantly enhances recrystallization-driven grain refinement, whose effect dominates over cooling-rate influences, ultimately yielding the finest grain size in the middle region. As for the bottom region, though influenced by both heat dissipation from the middle microstructure and interlayer thermal cycling, its proximity to the substrate creates superior heat dissipation conditions. The substrate actively absorbs both solidification heat from the bottom layer and a portion of interlayer thermal energy, producing higher cooling rates than the top/middle regions. Consequently, the bottom microstructure exhibits a smaller average grain size than the top region, yet a larger average grain size than the recrystallization-refined middle region due to the latter’s dominant grain refinement mechanism.

### 3.2. Mechanical Properties

**Figure 7:**
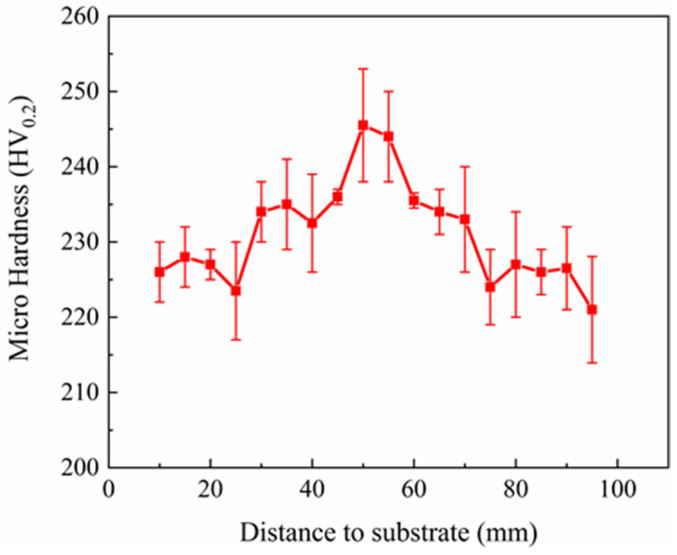
Hardness distribution of 2209 DSS in height direction.

The hardness data of different regions are shown in Figure 7. The average hardness values at the bottom, middle, and top sections along the height direction reached 230.27 HV, 238.57 HV, and 225.55 HV, respectively. The middle section exhibited the highest average hardness, while the top section showed the lowest. The difference in average hardness between the top and middle sections did not exceed 15 HV, and the overall maximum hardness difference throughout the entire structure was also within 30 HV. Fluctuations between the overall hardness and the average hardness at any given location were minor. Overall, the hardness distribution of the WAAM 2209 DSS was relatively uniform.

The bottom section, being close to the substrate, benefited from better heat dissipation conditions, resulting in a higher cooling rate. This rapid cooling provided insufficient time for ferrite to transform into austenite. Furthermore, the faster cooling rate led to a finer grain size within the microstructure, consequently contributing to the higher hardness observed at the bottom. The middle section experienced an increased proportion of recrystallization due to the interlayer thermal cycling effect. This recrystallization refined the grain structure within the microstructure, leading to the higher microhardness observed in the middle section. The top section had poorer heat dissipation conditions, resulting in a slower cooling rate. This allowed sufficient time for ferrite to transform into austenite. Consequently, the grain size at the top was coarser, resulting in the lowest hardness observed in the top section microstructure.

**Figure 8:**
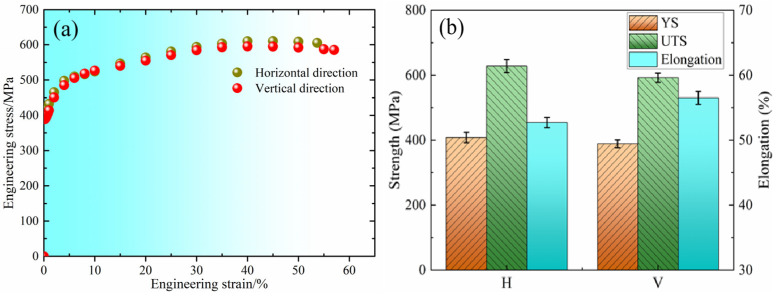
Mechanical properties of different region: (**a**) engineering stress–strain curves; (**b**) mechanical property parameter values (H, horizontal direction; V, vertical direction).

The mechanical properties of WAAM 2209 duplex stainless steel in different regions are shown in Figure 8. Figure 8a shows the engineering stress–strain curve, and Figure 8b presents the specific mechanical property parameter values. The ultimate tensile strength (UTS) and yield strength (YS) in the horizontal direction are 610 MPa and 408 MPa, respectively, while in the vertical direction they are 593 MPa and 389 MPa, respectively. It can be seen that the mechanical properties in the horizontal direction are superior to those in the vertical direction. The reason is that the sampling section of the horizontal tensile specimen essentially spans only two to three layers of deposited metal. The deposited material in this direction exhibits better tensile and yield strength because the deposition during the wire arc additive manufacturing process is essentially continuous, and the microstructural composition within a single layer is relatively uniform; furthermore, the microstructure and properties of adjacent layers are similar. Additionally, the microstructural characteristics between layers are essentially similar. Consequently, the microstructure of the deposited material is relatively dense in its orientation, leading to better tensile and yield strength. In contrast, the vertical tensile specimen spans a significant height through multiple deposited layers. Within this multilayer buildup, remelted zones and non-remelted zones alternate between different layers. The microstructure varies throughout the entire steel, and compositional differences exist between layers. Furthermore, interlayer thermal cycling effects occur during the arc processing. The presence of these factors results in lower yield strength and tensile strength.

Table 4 lists the tensile properties from this study along with those of selective laser melting (SLM) duplex stainless steel alloys and cast duplex stainless steels with similar chemical compositions [28,29]. Compared to the SLM DSS alloy and cast DSS, the coupons tested of the WAAM 2209 DSS in the current research demonstrated better comprehensive mechanical properties, namely a higher strength–ductility product. This is mainly attributed to the favorable microstructural features of the WAAM 2209 DSS.

**Figure 9:**
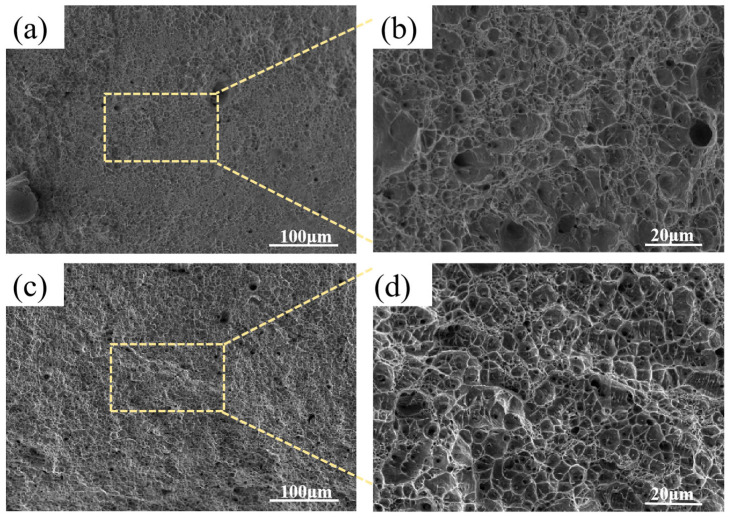
Fracture patterns of tensile components at different regions: (**a**,**b**) horizontal direction, (**c**,**d**) vertical direction.

Figure 9 displays the fracture modes of horizontal and vertical tensile specimens of WAAM 2209 duplex stainless steel. The dimples in both horizontal and vertical directions are predominantly equiaxed dimples, indicating a ductile fracture mode [23]. The horizontal direction exhibits fewer dimples than the vertical direction, with uneven dimple sizes and an overall size larger than those in the vertical direction. This suggests that the material possesses less ductility in the horizontal direction than in the vertical direction.

Based on the above analysis, the interpass thermal cycling experienced by the middle region of the WAAM 2209 steel deposit promotes recrystallization. This recrystallization leads to significant grain refinement and a reduction in low-angle dislocation boundaries within those grains. This refined microstructure, dominated by high-angle grain boundaries acting as strong obstacles to dislocation motion, is the primary reason for the higher strength (both YS and UTS) observed in the horizontal testing direction, which aligns with this strengthened microstructure within the deposition layers.

Conversely, the vertical testing direction exhibits higher ductility. This anisotropy in ductility is likely influenced by the inherent layered nature of the WAAM process, including the alignment of columnar grains along the build direction and the presence of inter-layer boundaries. These features provide paths or mechanisms that accommodate more plastic deformation before fracture when loaded parallel to the build direction, even though the average microstructure might be slightly coarser in the regions traversed (top/bottom). The dominant strengthening mechanisms (grain refinement) have a larger negative impact on the ductility in the horizontal direction compared to the vertical direction where grain morphology and layer effects favor elongation.

## 4. Conclusions

This study employed OM, SEM, and EBSD techniques to analyze the microstructural evolution and mechanical properties across multiple sections of WAAM 2209 duplex stainless steel fabricated via CMT-based arc rapid manufacturing technology. The principal conclusions are as follows and these findings establish a foundation for tailoring WAAM processing strategies to achieve balanced strength–ductility in duplex stainless steels for marine and industrial applications.

(1) The build height exhibits distinct phase evolution due to cooling rate variations. The bottom region (high cooling rate) develops elongated austenite with WA; the middle region (interlayer thermal cycling) achieves the finest grains (austenite: 4.16 μm, ferrite: 3.18 μm) and highest recrystallization fraction (∼50%), enhancing hardness (238.6 HV); and the top region (slow cooling) forms coarse blocky austenite. Secondary austenite (γ_2_) in interlayer zones shows Cr/Ni partitioning, increasing susceptibility to microsegregation.
(2) Horizontal specimens exhibit superior tensile strength (UTS: 610 MPa vs. vertical: 593 MPa) due to uniform intralayer microstructures and minimal interlayer defects. Conversely, vertical specimens display higher ductility, facilitated by columnar grain alignment (<001>//Z texture in ferrite, <101>//Z in austenite) and layered boundaries accommodating plastic deformation. Fractography confirms ductile failure with equiaxed dimples, finer in vertical samples.
(3) CMT-WAAM enables dense, high-performance DSS2209 components with moderate hardness uniformity (Δ < 30 HV). Interlayer thermal cycling critically governs recrystallization and grain refinement, while substrate proximity accelerates bottom-layer cooling.

## Figures and Tables

**Table 1:** Chemical composition (in wt%) of as-deposited.

Cr	Ni	Mo	Mn	Si	N	O	Fe
22.7	9	3.2	0.9	0.8	0.13	0.03	Balance

**Table 2:** EDS analysis of WAAM 2209 DSS.

Position	Phase	Alloying Element wt (%)		
		Cr	Ni	Mo
Top	α	24.2 ± 0.2	5.4 ± 0.4	3.7 ± 0.1
	γ	21.7 ± 0.1	5.8 ± 0.4	3.5 ± 0.1
	γ_2_	21.0 ± 0.2	7.2 ± 0.5	3.2 ± 0.2
Middle	α	23.7 ± 0.1	5.0 ± 0.4	3.9 ± 0.1
	γ	22.6 ± 0.1	5.6 ± 0.4	3.7 ± 0.2
	γ_2_	21.7 ± 0.2	7.8 ± 0.5	3.2 ± 0.2
Bottom	α	25.2 ± 0.1	5.5 ± 0.4	4.5 ± 0.1
	γ	22.4 ± 0.2	5.8 ± 0.4	3.6 ± 0.2
	γ_2_	22.0 ± 0.2	7.6 ± 0.5	3.1 ± 0.2

**Table 3:** EBSD correlation data for austenite (γ-Fe) and ferrite (α-Fe).

Position		XOZ			YOZ			XOY		
		Bottom	Middle	Top	Bottom	Middle	Top	Bottom	Middle	Top
γ-Fe	Grain Size (μm)	4.5 ± 0.4	4.0 ± 0.5	4.6 ± 0.4	4.0 ± 0.5	4.2 ± 0.4	5.1 ± 0.4	4.9 ± 0.5	4.2 ± 0.5	5.2 ± 0.4
	Fraction (%)	66.7 ± 3.0	66.4 ± 3.0	66.9 ± 3.0	59.1 ± 3.0	58.7 ± 3.0	62.1 ± 3.0	59.9 ± 3.0	57.8 ± 3.0	60.8 ± 3.0
	LAGB	87.2 ± 2.0	85.6 ± 2.0	85.0 ± 2.0	89.1 ± 2.0	89.5 ± 2.0	90.7 ± 2.0	94.4 ± 1.5	85.6 ± 2.0	93.6 ± 1.5
	HAGB	12.8 ± 2.0	14.4 ± 2.0	15.0 ± 2.0	10.9 ± 2.0	10.5 ± 2.0	9.3 ± 2.0	5.6 ± 1.5	14.4 ± 2.0	6.4 ± 1.5
	Recrystallized	16.2 ± 2.5	17.4 ± 2.5	23.3 ± 3.0	17.9 ±2.5	26.0 ± 3.0	17.3 ± 2.5	10.9 ± 2.5	30.8 ± 3.0	10.3 ± 2.5
	Substructured	50.2 ± 3.0	53.9 ± 3.0	58.9 ± 3.0	42.7 ± 3.0	49.6 ± 3.0	49.3 ± 3.0	15.2 ± 2.5	44.3 ± 3.0	14.6 ± 2.5
	Deformed	33.6 ± 3.0	28.7 ± 3.0	17.8 ± 2.5	39.3 ± 3.0	24.4 ± 3.0	33.3 ± 3.0	73.8 ± 3.0	24.9 ± 3.0	75.1 ± 3.0
α-Fe	Grain Size (μm)	3.4 ± 0.4	3.1 ± 0.3	3.7 ± 0.4	3.6 ± 0.4	3.2 ± 0.3	3.8 ± 0.4	3.5 ± 0.4	3.2 ± 0.3	4.1 ± 0.4
	Fraction (%)	33.3 ± 3.0	33.6 ± 3.0	33.1 ± 3.0	40.9 ± 3.0	41.3 ± 3.0	37.9 ± 3.0	40.1 ± 3.0	42.2 ± 3.0	39.2 ± 3.0
	LAGB	97.4 ± 1.0	96.7 ± 1.0	94.9 ± 1.5	96.4 ± 1.0	97.9 ± 1.0	96.1 ± 1.0	98.3 ± 0.8	93.1 ± 1.5	98.0 ± 0.8
	HAGB	2.6 ± 1.0	3.3 ± 1.0	5.1 ± 1.0	3.6 ± 1.0	2.1 ± 1.0	3.9 ± 1.0	1.7 ± 0.8	6.9 ± 1.0	2.0 ± 0.8
	Recrystallized	25.9 ± 3.0	36.1 ± 3.0	34.7 ± 3.0	25.0 ± 3.0	25.4 ± 3.0	29.7 ± 3.0	10.0 ± 2.5	26.6 ± 3.0	14.7± 2.5
	Substructured	54.0 ± 3.0	39.4 ± 3.0	43.8 ± 3.0	52.9 ± 3.0	49.7 ± 3.0	47.9 ± 3.0	10.4 ± 2.5	45.2 ± 3.0	17.7 ± 2.5
	Deformed	20.1 ± 3.0	24.5 ± 3.0	21.5 ± 3.0	22.1 ± 3.0	24.9 ± 3.0	22.5 ± 3.0	79.6 ± 3.0	28.2 ± 3.0	67.6 ± 3.0

**Table 4:** Comparison of tensile properties between WAAM 2209 duplex stainless steel and those fabricated by other processes.

Process	Specimen Location	Ultimate Tensile Strength (MPa)	Yield Strength (MPa)	Elongation (%)	Ultimate Tensile Strength × Elongation (GPa·%)	References
SLM DSS as-built condition	/	940	/	12	11.3	[28]
CAST DSS	/	695	450	25	17.4	[29]
CMT-WAAM 2209DSS as-built condition	horizontal direction	610	408	53	32.3	Present work
	vertical direction	593	389	57	33.8	

## Data Availability

The original contributions presented in this study are included in the article. Further inquiries can be directed to the corresponding author.
